# Providing Dignified Palliative Care Services in Liberia

**DOI:** 10.5334/aogh.2590

**Published:** 2019-10-15

**Authors:** Sonpon Blamo Sieh, Chinnie Vicky Miller Sieh, James Desmond, Catherine C. Machalaba

**Affiliations:** 1 Home of Dignity, Inc, Yarbah’s Town, Virginia, St. Paul District, LR; 2 Liberia Chimpanzee Rescue and Protection, Charlesville, LR; 3 Policy Advisor and Research Scientist, EcoHealth Alliance, NY, New York, US

## Abstract

**Background:**

Liberia faces a critical shortage of palliative care services, particularly for persons with advanced-stage HIV/AIDS, tuberculosis, diabetes, and cancers. Access to healthcare services is especially limited in rural areas, along with a lack of supportive social and economic resources. Home of Dignity (HoD) Health Center was established in 2013 in Yarbah’s Town to fill a last-option palliative care gap. The mission emphasizes patient wellbeing and worth. HoD integrates health, agriculture, and education on-site for immediate medical needs, broader sustainable development, and reducing disease-associated stigma in local communities.

**Objective:**

We aimed to describe the Center’s integrated approach and conduct a descriptive analysis of the HoD patient population.

**Methods:**

We reviewed patient characteristics (sex, age distribution, mobility status, and CD4 count on arrival) and outcomes (survival rate and community reintegration) for patients with HIV seeking care at the Center between 2013–2017.

**Findings:**

Of 182 patients (ages 3 months–50 years), over half arrived to the facility bedridden and over 82% had CD4 counts between <100–350. Of the 182 patients, 66% survived, 27% died, and 7% were lost to follow-up. Of surviving patients, 90% were successfully reintegrated into their communities. The clinic also served over 365 chronically ill patients that had been rejected by other health providers during the 2014–2015 Ebola outbreak.

**Conclusions:**

The Center is providing last-option palliative care services in the country. As a trusted healthcare center, patients also seek care for acute conditions, resulting in unanticipated resource demands. HoD’s experience underscores the need for development of training programs for medical professionals, supply chains, community outreach, and resourcing channels to ensure adequate and sustainable service provision for hospice and palliative care services and reduce stigma in the country. There is an urgent need to invest in holistic palliative and overall healthcare services in Liberia.

## Introduction

Palliative care is widely under-served in Africa, meeting <5% of need for the continent’s population, despite its recognition as a humanitarian need by the World Health Organization (WHO) [1]. At the same time, the rise in non-communicable diseases is expected to increase chronic care demands. This introduces major service delivery challenges, especially in settings where access to healthcare services is already limited. As of 2016, there were no formally-recognized national palliative care policies, service delivery, and medical education in many countries in West Africa, including Liberia [2,3,4].

Liberia is a small coastal country with a population of 4.6 million and was one of the three countries primarily impacted by the 2014–2015 West Africa Ebola epidemic. Healthcare services remain extremely limited, partly due to residual impacts of civil war and extremely low health worker density (e.g., 0.028 physicians and 0.457 nurses and midwives per 1,000 population in 2010) further exacerbated by losses of frontline healthcare providers from Ebola [5,6]. Most of the country’s specialized care is concentrated in Monrovia, the capital city. No hospice-palliative care activities were identified in 2011 [7]. While prevalence of HIV/AIDs is lower than other African regions, the country has not benefitted from investments through the US President’s Emergency Plan for AIDS Relief that improved palliative care capacity elsewhere in the continent [8]; stigma is common and there is low screening and poor management of the disease. There are particularly severe service gaps in care provision for patients with advanced-stage HIV/AIDS, Tuberculosis (TB) and cancers. Additionally, incidence of non-communicable diseases is increasing in Liberia; a recent study conducted by the National Public Health Institute of Liberia found a rising prevalence of diabetes or pre-diabetes, particularly in urban populations [9]. The population faces low health literacy, nutritional security, and employment [10].

Rural populations and natural resources are closely linked in Liberia, including dependencies for food, water, and often livelihoods. Only 17% of the population has access to basic sanitation [11]. Thus, a “One Health” approach that recognizes the integral links between the health of humans, animals, and the environment is promoted by the Government [12,13], particularly to increase prevention, detection, and resilience to disease threats post-Ebola. This paper highlights efforts of a health center providing the first dignified palliative care services in Liberia over the past five years, including integration of multi-sectoral One Health strategies to address the social and environmental determinants of health, maximize patient dignity, and advance sustainable development.

### An Integrated Approach

**Figure 1:**
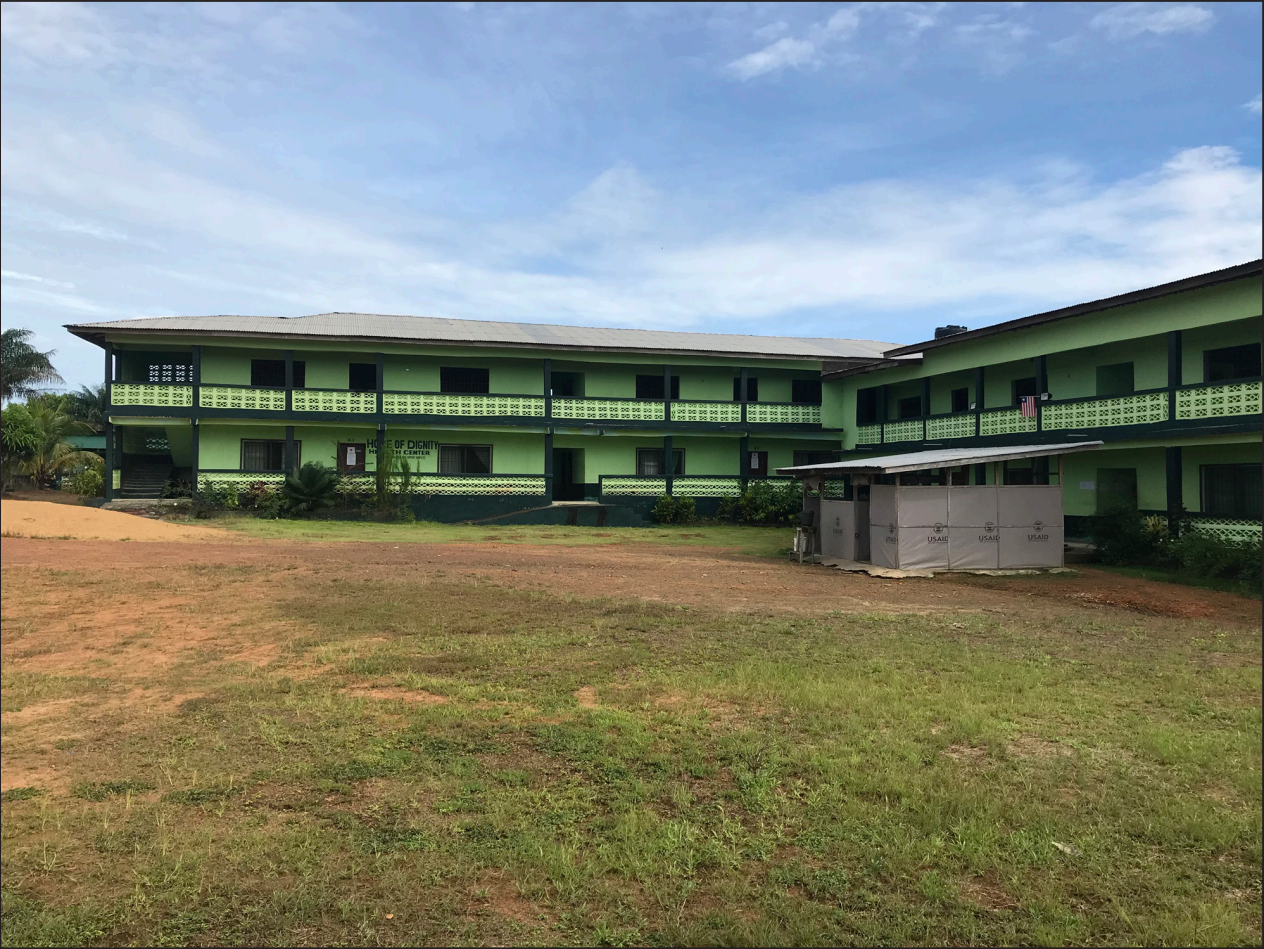
Patient ward at Home of Dignity Health Center.

The Home of Dignity (HoD) Health Center was established in 2013 in Yarbah’s town, Virginia, St. Paul District in Montserrado County, a rural community approximately forty-minutes outside of the Freeport of Monrovia. HoD’s mission is to provide high-quality, reliable and compassionate care, treatment and support services for the dignity of life (Figure 1). On-site healthcare providers cover the center’s inpatient beds and outpatient services.

Initial support to build the center came from the Grant Assistance for Grassroots Human Security Project through the government of Japan in 2012. Following the 2014–2015 Ebola Virus Disease Outbreak, the WHO Liberia Office provided financial support for rehabilitation and construction of the upper floor of the in-patient center in 2015. HoD’s core operations have largely been sustained via local resources (e.g. from the United Methodist Church Men Fellowship, Monrovia District and contributions from the founders), private donors, and volunteer staff. Medications for the treatment of HIV/AIDs and TB are provided through the Ministry of Health with financial support from the Global Fund.

**Figure 2:**
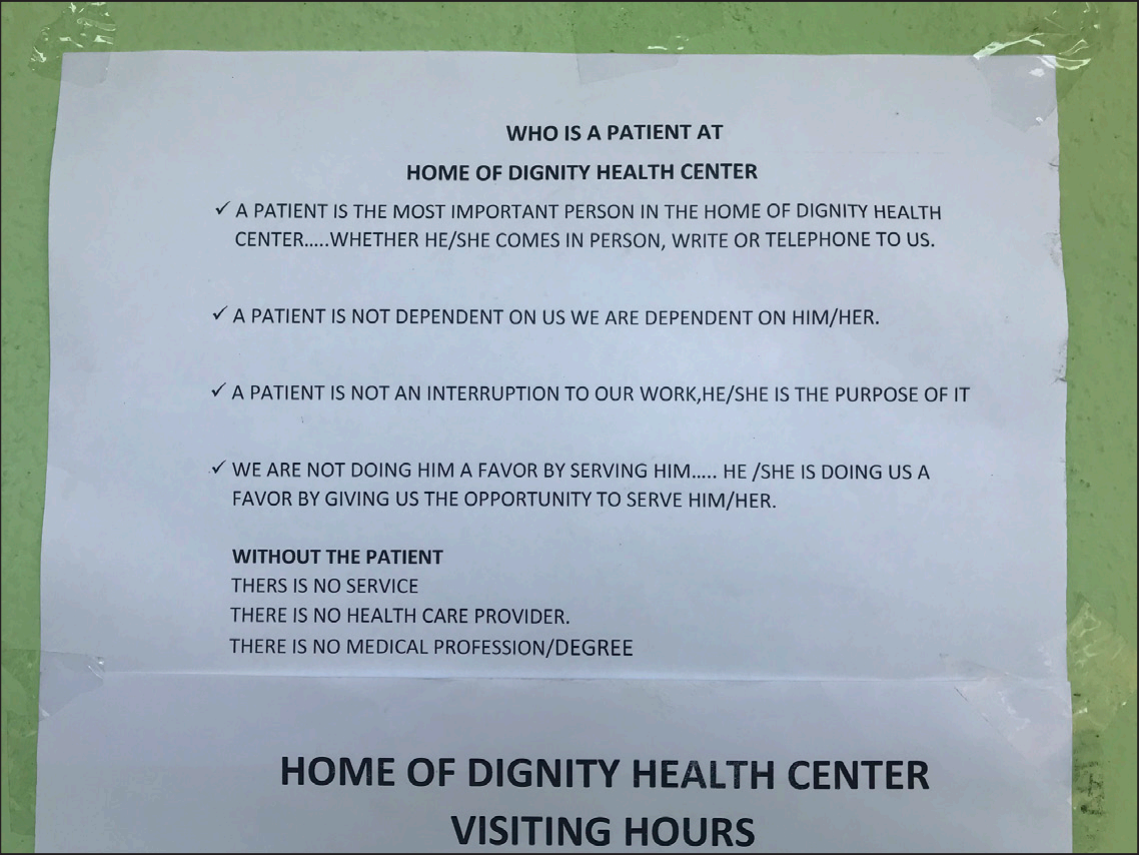
Home of Dignity Mission statement.

**Figure 3:**
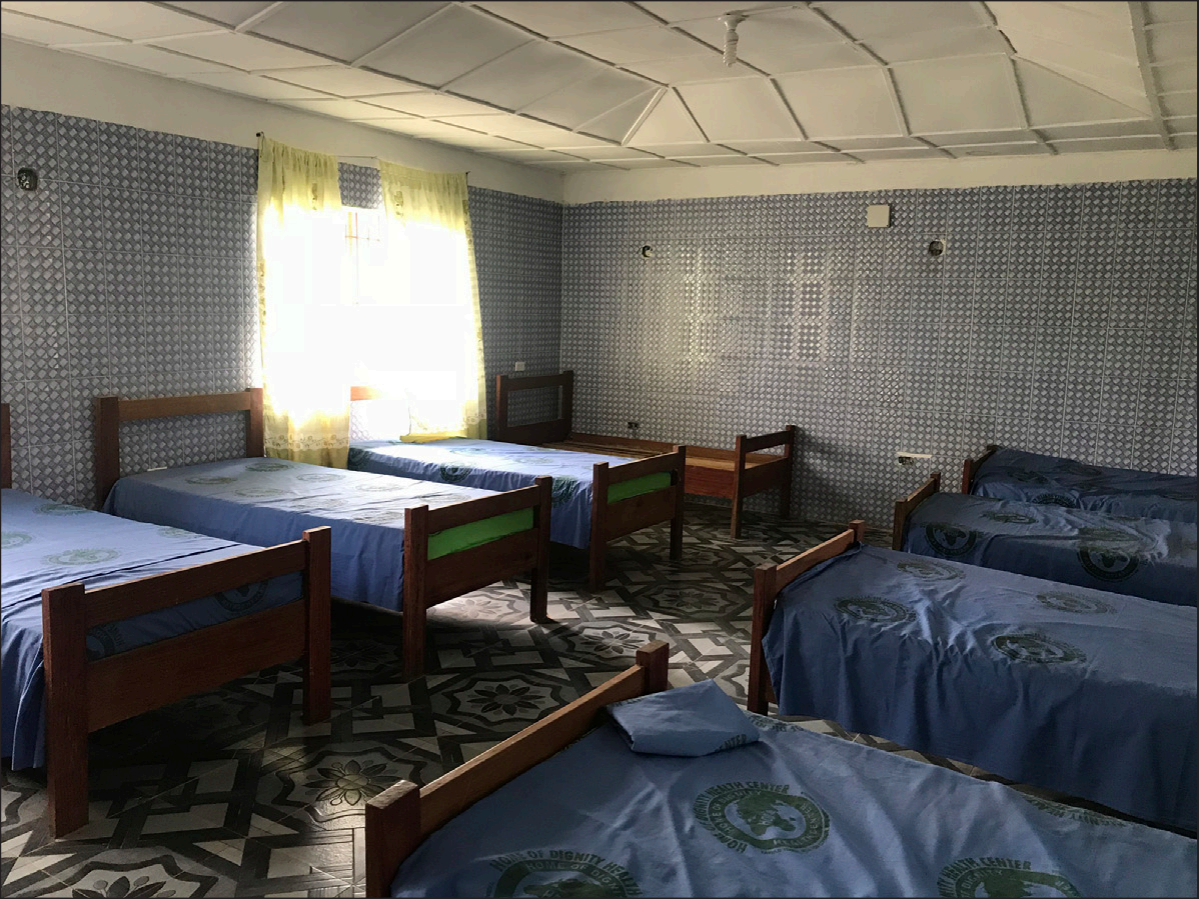
Patient care room.

**Figure 4:**
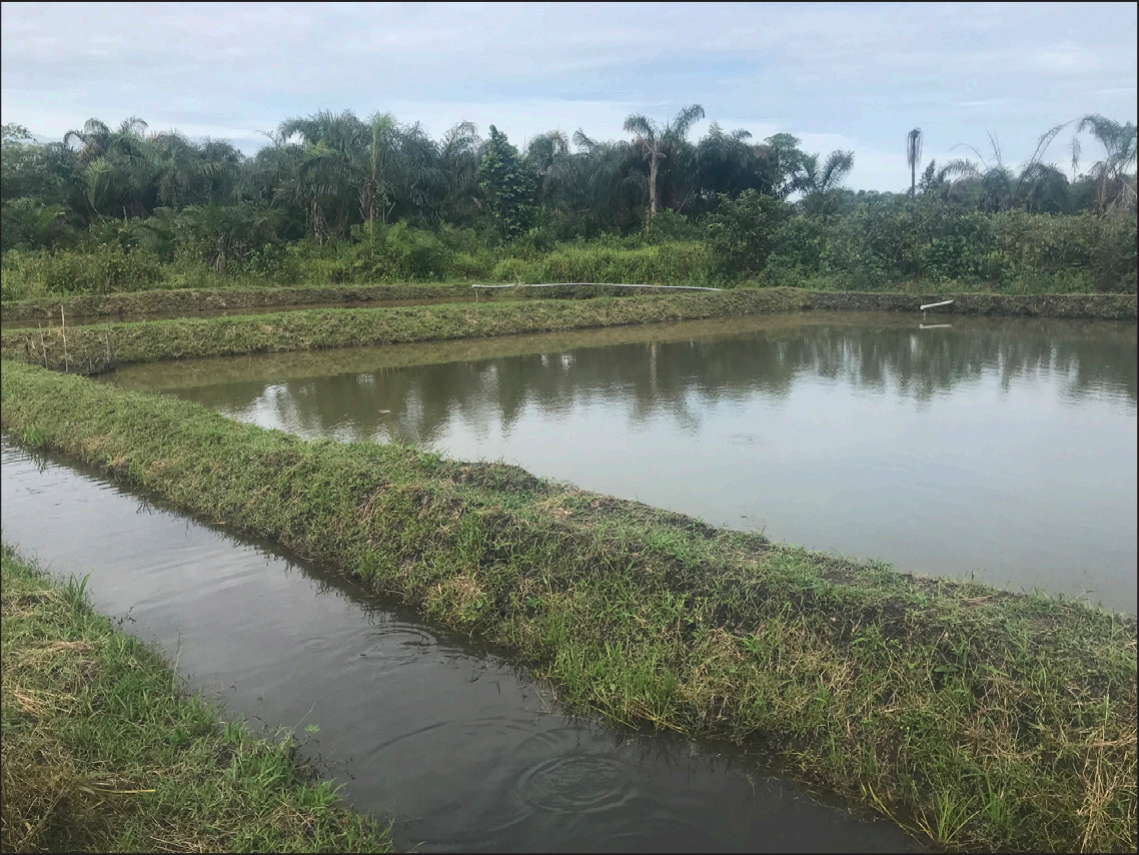
The on-site aquaculture farm was developed to provide a source of nutrition and revenue to support the Center’s operations.

Consistent with a “One Health” approach, the organization was expanded to be multi-sectoral and currently provides services in the areas of Health, Agriculture/Aquaculture, and Education for Sustainable Development, and officially registered in 2017. Crops (cassava, palm, and peppers) are grown on-site. In 2018, an aquaculture farm was established in consultation with the Ministry of Agriculture to provide nutrition for patients and generate revenue. Patients can walk freely around the property, which has outdoor seating areas. The Center’s mission is posted throughout the facility to be highly visible to patients, care providers, and visitors, noting that the patient is not an interruption to providers’ work but rather “the purpose for which they are available” (Figures 1, 2, 3, 4).

## Methods

We analyzed patient characteristics (age, gender, mobility status, and CD4 count on arrival) and outcomes (survival rate and community reintegration) for patients with HIV seeking care at the Center from December 1, 2013 through December 31, 2017. Information had been collected as part of routine patient record keeping; these were reviewed on-site under supervision of the clinical care team, with all patient identifiers removed. Data was analyzed in Excel. Information reported on HIV prevalence was previously supplied by the Center’s clinical team. We also reviewed overall patient numbers to assess overall demand, including patients presenting during the Ebola epidemic.

## Results

The Center has provided free or subsidized long-term care, including treatment and supportive hospice services, to more than 300 chronically ill patients by the end of October 2018. Persons seeking care have spanned those with HIV, TB, malaria, cancers, diabetes, and victims of sexual and gender-based violence. In a review of the 182 patients with HIV from 2013 through 2017 (Table 1), the majority of patients were female (68%) and of adult age (82%) (defined as age 15+). Patient age at time of intake ranged from 3 months to 50 years. Patients arrived via referral from 11 other hospitals or clinics in Liberia (56%) or directly to the Center (44%).

**Table 1:** Characteristics and Outcomes of Patients, Home of Dignity Health Center, December 1, 2013–December 31, 2017.

Indicator	No. (%) of Patients n = 182

*Sex*	
Female	124 (68.13)
Male	58 (31.87)
*CD4 Count on Arrival*	
<100	99 (54.40)
100–350	51 (28.02)
351–500	15 (8.24)
501–1000	15 (8.24)
1001–1500	2 (1.10)
*Arrival Status*	
Bedridden	99 (54.40)
Ambulatory*	66 (36.26)
Other^†^	17 (9.34)
*Outcome*	
Survived	120 (65.93)
Deceased	50 (27.47)
Lost to follow up	12 (6.59)
*Community Reintegration of Surviving Patients*	
Successful	108 (90.00)
Unsuccessful^§^	12 (10.00)

* Ambulatory represents persons able to walk but brought into the facility by relative or family member for treatment or illness with consent. ^†^ Other represents persons who willingly visit the facility and request getting tested for their HIV and/or TB status. ^§^ Neglected or rejected by family/relatives.

By the end of 2017, over 82% of the 182 patients arriving at HoD with chronic conditions with very poor prognosis were observed with CD4 cell counts ranging from <100–350. Of the 50 patients who died, CD4 count on arrival was <100 in 88% (44 patients), and 64% of patients who passed away had an average duration of stay at the clinic (representing a proxy for duration of exposure and adherence to ARTs) of six month or less prior to death. Other associated conditions observed at death included Anemia (58%), Kaposi Sarcoma (20%), Herpes Zoster (10%), Extra Pulmonary TB (6%), Oral Candidiasis (4%), and Heart Failure (2%). Of the patients, 54.4% were brought bedridden and 36.3% arrived at HoD ambulatory by relatives. Due to improved case management of conditions, survival rate was 73% during the period, excluding patients lost to follow up. Of patients undergoing treatment for HIV/AIDS, the most common treatment has been TDF/3TC/EFV (46% of patients) and AZT/3TC/NVP (28%), followed by other ART combination protocols. Fifteen patients (8%) refused treatment on arrival or were not prescribed ARTs. Of the 32 children (ages 0–14) brought to the facility, four died early into arrival. Of 28 who were treated and receiving care, the majority (96%) were placed on antiretroviral therapy (ART); included in this population were pregnant women <15 years of age.

Community engagement and health education are cornerstones to breaking the barrier of stigma and discrimination. Community reintegration was tracked as an outcome indicator (here referring to a qualitative, patient-reported dichotomous [yes/no] measure of acceptance attitude of family members, relatives, and friends of people impacted by HIV and other chronic conditions brought to the facility who recovered and linked back with their families and relatives at community level without rejection, neglect, stigma, and discrimination). Of the patients who recovered but were given routine schedules to attend the clinic (e.g., for medication refill), were sent to their respective homes, and re-connected to their families, 12 of these persons were rejected by families, relatives, and friends due to their HIV status or other medical conditions. There were also two cases of discordant couples among the 12 patients who were rejected and lost or had broken relationships and returned to the facility. Five of the patients who died were neglected by family members, relatives, and friends and through the help of the local police authority and Home of Dignity their bodies were laid to rest.

During the period, 90% of those who survived were reintegrated into communities with their families, while 10% were neglected/rejected by relatives. Many currently live within the HoD compound and are provided accommodation and feeding; several serve as peer educators there for economic empowerment. HoD plans to provide community health education to the general population with a focus on very sick and/or chronically ill persons needing care and support services, using screening for HIV and TB as entry points.

For World AIDS Day the Center conducts outreach including free HIV screening to raise awareness about the benefits of prevention, early detection and treatment of the disease to reduce the heavy stigma it carries in the country, reaching 4,847 persons to date. Country-wide general HIV prevalence of 2.1% was recorded in the last Demographic and Health Survey [14]. Free testing by HoD since 2013 indicates a prevalence of 3.9%, suggesting the Center is reaching persons not otherwise formally connected the healthcare system.

During the 2014–2015 Ebola Virus Disease Outbreak, HoD also provided free services to >365 chronically ill people (non-Ebola-suspected) that had been rejected by healthcare workers and health facilities based on fear of infection and the overwhelmed healthcare system.

## Discussion

The importance of establishing and maintaining access to routine healthcare services is increasingly apparent in Liberia and neighboring countries; for example, an estimated 1,500 additional indirect deaths in the country were attributed to disruptions in diagnosis and treatment for malaria, TB, and HIV during the outbreak [15]. A recent review and meta-analysis also estimated the impact of the West Africa Ebola outbreak as a reduction of 18% of non-Ebola-associated health service utilization in Guinea, Liberia, and Sierra Leone, noting that this was on top of already-insufficient utilization prior to the epidemic [16]. Our findings suggest that Home of Dignity is reaching patients with otherwise unmet needs for chronic conditions, as demonstrated by the severity of their conditions on arrival (CD4 count and bedridden status and the lack of existing treatment), while also serving wider demand from patients without access to routine medical services during disease emergencies.

Recognition of need for palliative care capacity is advancing in the country. In October 2018 the Ministry of Health hosted an intensive training in palliative healthcare in collaboration with the Uganda-based Palliative Health Care Association [17]. Although liquid morphine or opioids were not available at the facility during the period under review, the Center’s advocacy has helped to advance pain management offerings in the country, including changes to drug regulations allowing morphine use at in-patient healthcare centers. However, there is limited familiarity among providers on proper use in addition to the overall supply chain issues in Liberia that currently hinder consistent access to affordable and safe medicines.

Beyond direct care service provision, Liberia faces several infrastructure and socio-economic challenges that affect health status, testing and treatment access, and treatment compliance outside of hospital and clinic walls. These are issues generally broadly appreciated in public health and health service delivery in the country, but not raised specifically in the context of palliative care for patient recovery and initial treatment seeking. We looked at patient characteristics and outcome indicators to provide an initial profile of patients and their needs to identify areas of further service delivery and research. In particular, the concept of overcoming stigma and implications for successful patient reintegration into communities, by empowering patients and showing improved (and often visible) health status and treatment success, could be an important area for further study. Examining and comparing staff, patient, and community member perceptions related to treatment and reintegration barriers could be highly informative, especially if paired or followed up with low-resource intervention testing in a systematic way across different settings. For example, peer health educators may potentially help advance health literacy and pair treatment needs and services. The high percentage of adult females of patients served indicates that special attention may be needed to reach this population; this may be reflective of the higher HIV prevalence in women than men in the country [18], or other diagnostic and treatment access determinants that influence the severity of disease when they arrive for care.

While we focus on palliative care as a major, largely unaddressed need in the country, Liberia faces many areas of limited capacity which addressing in coordinated fashion may help to reduce overall disease burden and demand for health services. In addition to palliative care services, the range of infectious and non-infectious health threats in the country, the socio-economic factors that influence risk and outcomes, and the demand seen from patients with non-chronic conditions (e.g. those needing routine healthcare services during the Ebola epidemics) raise opportunities for strengthening capacity in integrated ways that promote gains in multiple disciplines and sectors. For example, HoD’s holistic model has embraced a One Health approach from the onset, including designating space for an animal health laboratory in the design and building process. This is relevant as the country has an extremely limited veterinary medicine workforce and animals are not routinely vaccinated or surveilled for rabies and other preventable zoonotic diseases. While we did not assess zoonotic disease prevalence in patients, information on animal exposures and animal diseases may help to identify potential causes of co-morbities [19]. Developing this capacity will also support animal health on-site, as the Center presently has poultry rearing and is in the process of building swine operations for nutrition and income resources. This is ultimately envisioned to provide hands-on skills training for livestock production, with long-term plans for an education center for patients and community members to address critical workforce deficiencies, and reduce stigma through increased health literacy and visibility of health services. In low-resource settings with severe capacity gaps in multiple sectors, integrated approaches like this may contribute to greater mainstreaming of palliative care while contributing to broader sustainable development and community resilience.

## Conclusions

The Center has far exceeded the planned scope of care, resulting in higher case load and more clinically diverse conditions than expected. Additionally, emergency care centers have brought patients who cannot afford treatment post-discharge, and some patients have traveled from far (for example, from Guinea), in part to avoid stigma of seeking local care. The Center’s holistic attention to patients’ medical, housing, and nutrition needs and overall dignity provides a novel approach to promote rehabilitation and effective disease management. The demand reinforces that the center is providing last-option care; however, this has resulted in high unanticipated resource needs.

The Center faces persistent infrastructure issues common in Liberia, including inconsistent supply of pharmaceuticals, including some essential medicines. Electricity is provided from an on-site generator, although long-term plans include solar power generation. Flooding makes physical access challenging, and the site’s greenhouse was damaged by heavy rains.

HoD’s experience highlights needs and opportunities for similar organizations and country capacity. First, finding the right mix of local civil society organizations for achieving better health and social justice outcomes by using One Health approaches with local and international partners is needed to support resilient health systems for underserved populations with limited public health infrastructure, particularly after the Ebola epidemic. To ensure long-term capacity and sustainability, the country must develop a national curriculum for palliative and hospice care services and quality of care. For successful for universal health coverage, national government and development partners must widen engagement for innovative service delivery in palliative and hospice care to fast track action towards the 90-90-90 targets for HIV treatment. This can be supported by strengthened diagnostic capacity, public-private partnerships, and media and community engagements to create sensitization and awareness.
